# Characterization of metal binding sites onto biochar using rare earth elements as a fingerprint

**DOI:** 10.1016/j.heliyon.2018.e00543

**Published:** 2018-03-01

**Authors:** Olivier Pourret, David Houben

**Affiliations:** UniLaSalle, AGHYLE, Beauvais, France

**Keywords:** Chemistry, Geochemistry, Environmental science

## Abstract

The ability of biochar to immobilize metals relies on the amount of functional groups at its surface but the contribution of each functional groups (e.g. carboxylic, phenolic) to metal bonding is poorly known. Using a new approach based on previous works on rare earth element (REE) interactions with humic substances, we aim at elucidating the relative contribution of these binding sites to metal sorption under various conditions (i.e. pH and ionic strengths, IS). Using batch experiments, REE sorption onto biochar was analyzed from pH 3 to 9 and IS 10^−1^ mol/L to 10^−3^ mol/L. Rare earth element patterns show a Middle REE (MREE) downward concavity at acidic pH and low ionic strength. These patterns are in good agreement with existing datasets quantifying REE binding with humic substances. Indeed, the MREE downward concavity displayed by REE-biochar complexation pattern compares well with REE patterns with various organic compounds. This similarity in the REE complexation pattern shapes suggests that carboxylic groups are the main binding sites of REE in biochar. Overall, our results indicate that the strength of the metal bonding with biochar increases when pH and IS increase, suggesting that biochar is more efficient for long-term metal immobilization at near neutral pH and high ionic strength.

## 1 Introduction

Biochar, the carbon-rich product of biomass pyrolysis, has sparked a broad interest due to its attractive potential for improving soil fertility, carbon sequestration and soil remediation (Beesley et al., 2011; Houben et al., 2013a; Lehmann and Joseph, 2009). Surface functional groups play an important role in the application of biochar as a functional material, especially for the immobilization of toxic metals (Chen et al., 2015; Liu et al., 2015). For example, using biochars produced at 400 and 700 °C from rice straw and cattle manure, Qian and Chen (2013) found that aluminum (Al) binding onto biochar was predominantly due to complexation of Al with the hydroxyl and carboxyl group at the surface of biochar particles. Similarly, the sorption of copper (Cu) and lead (Pb) was found to be directly correlated with the amount of oxygen-containing carboxyl, hydroxyl, and phenolic functional groups at the surface of biochars produced at various temperatures (200–800 °C) from cottonseed hulls (Uchimiya et al., 2011). Understanding biochar surface reactivity is therefore a prerequisite to its application in soils. Spectroscopic approaches including Fourier transform infrared spectroscopy (FTIR) and nuclear magnetic resonance spectroscopy have been frequently employed to characterize the functional groups of biochar (Brewer et al., 2011; Keiluweit et al., 2010; Zhao et al., 2013). However, these techniques do not allow determining to what extent each functional group contributes to metal binding under various conditions. Previous research on humic substances has shown that metal binding to carboxylic and phenolic sites was strongly dependent on pH and ionic strength in the aqueous phase (Guo et al., 2008; Ravat et al., 2000; Tipping, 2002). Characterization of humic substances has also revealed that carboxylic groups generally exhibit low metal affinity sites, while phenolic groups host high affinity sites (Benedetti et al., 1995; Kinniburgh et al., 1999). Unraveling the relative contribution of carboxylic and phenolic groups to metal binding by biochar under various pH and ionic strengths is therefore crucial to gain insight into the strength of association between metal and biochar and to predict metal interaction with biochar over a wide range of conditions.

An emergent approach for characterizing the surface reactivity of organic substances consists in analyzing the pattern of rare earth element (REE) sorption by these compounds (Pédrot et al., 2010). Rare earth elements are a group of seventeen elements presenting very similar and coherent chemical properties (Connelly et al., 2005). In this contribution, we will consider the fifteen lanthanides as REEs. Due to the unique properties of REEs, their partition coefficients (Kd; commonly used in estimating the potential sorption of dissolved metals by a solid phase) between humic substances and aqueous solution are highly sensitive to the heterogeneity of binding sites (i.e. the nature of the functional group involved) (Marsac et al., 2010; Sonke and Salters, 2006). As a result, specific Kd patterns arise depending on the nature of the major binding sites at the surface of humic substances. For example, log Kd pattern exhibits a medium REE (MREE) downward concavity when REE binding occurs at carboxylic sites (Pourret et al., 2007). On the other hand, REE binding to phenolic groups results in a log Kd pattern exhibiting a regular increase from light REEs (LREEs) to heavy REEs (HREEs) (Davranche et al., 2015; Marsac et al., 2010). More specifically, studies have revealed that LREEs are mainly bound by weak carboxylic sites at pHs ranging between 3 and 6, while HREEs are preferentially bound to strong multidentate sites at pH = 3 and to phenolic groups at pH = 6 (Marsac et al., 2011). These findings have raised the possibility to use REEs as a tool to determine which of the carboxylic, phenolic or chelate functional groups are implicated in the binding of cations to humic substances under various environmental conditions (Davranche et al., 2015; Marsac et al., 2011).

In the current study, a new approach based on previous works on REE interactions with humic substances, was developed to provide new insight into the nature of the sorption sites responsible for metal binding to biochar under various pH and ionic strengths.

## 2 Material and methods

### 2.1 Biochar characteristics

Biochar was obtained from VT Green (France) who uses an industrial pyrolysis reactor (Biogreen®, ETIA, France) and sewage sludge as feedstock. Operating conditions include a residence time in the reactor of 10 min and an end temperature of pyrolysis of 500 °C. The biochar was then rinsed with MilliQ water and ground to 0.05 mm particle size. The pH of the obtained biochar was 8.2 (biochar:water ratio of 1:10; w/v). Its electrical conductivity at 20 °C was 0.009 mS/cm and its cation exchange capacity was 4.6 mmol_c_/kg (cobalthexammine method) and its specific surface area was 19 m^2^/g (BET method). The biochar was further acid-rinsed with HNO_3_ at pH 2 to remove ash and avoid masking metal sorption by precipitation phenomenon and by metal complexation with inorganic ligands like carbonate (Houben and Sonnet, 2015). The PZC value of the studied biochar is 7.81, which is consistent with previous studies that evidenced an average value of 8.1 and a range from 6.2 to 9.6 (Lu et al., 2013). The biochar surface functional groups were measured with FTIR: 0.7 mg of biochar was gently mixed with 100 mg of KBr and the mixture were pressed into a pellet. FTIR spectrum of the biochar was recorded using a Nicolet iS10 spectrophotometer (Thermo Fisher Scientific), scanning from 4000 to 400 cm^−1^ at a resolution of 1 cm^−1^.

The following FTIR broad-band assignment was used to characterize the biochar (Jindo et al., 2014; Keiluweit et al., 2010): 3400 to 3410 cm^−1^, H-bonded O–H stretching vibrations of hydroxyl groups from alcohols, phenols, and organic acids; 2850 to 2950 cm^−1^, C–H stretching of alkyl structures; 1580 to 1590 cm^−1^, COO^−^ asymmetric stretching; 1460 cm^−1^, C–H deformation of CH_3_ group; 1280–1270 cm^−1^, O–H stretching of phenolic compounds; and 1000–1100 cm^−1^, bending of Si–O stretching. The sewage sludge-derived biochar from this study have comparable properties to those from previous studies (Lu et al., 2012).

### 2.2 Experimental setup description

All chemicals used in this study were of analytical grade, and all the experimental solutions used were prepared with doubly deionized water (MilliQ system, Millipore). REEs–biochar complexes were prepared in glassware containers previously soaked in 10% HNO_3_ for 48 h at 60 °C, then rinsed with MilliQ water for 24 h at 60 °C to remove all possible REE contamination sources. Synthetic REE solutions were prepared from nitrate REE standards (10 mg/L, Accu Trace Reference Standard).

Batch experiments were performed to study the pH and ionic strength effect on REE sorption by biochar as performed for humic acid (HA) by Pourret et al. (2007). REE (50 μg/L of each REE) and biochar (20 mg/L) were added together in solution, at various ionic strength (0.001 mol/L to 0.1 mol/L, adjusted by NaCl) and pH (from 3 to 9, adjusted by HCl and NaOH). Prior to addition of biochar, the pH of the solution was adjusted to near 4. The initial hydroxide and carbonate concentrations were considered negligible and concentrations of LnOH^2+^ and LnCO_3_^2−^ were therefore also considered negligible. The pH was measured with a Mettler Toledo EL 20 pH-meter equipped with a LE407 electrode having an accuracy of 0.01 pH units. Solutions were agitated at 120 rpm for 48 h (equilibrium time determined from preliminary kinetic experiments) at room temperature (20 ± 2 °C) to allow equilibrium and REES fractionation between aqueous solution and biochar suspension. Solution aliquots of 10 mL were sampled at the beginning of the experiment and at equilibrium state (i.e., after 48 h). REEs sorbed by biochar were separated from the remaining dissolved REE by filtration through 0.2 μm acetate cellulose filters.

To check possible sorption of inorganic REE species onto the membrane of the filter or onto tube walls, inorganic REE solutions of known REE concentration were filtered for each experimental conditions and monitored. More than 99% of the REEs present in the solution were recovered in the filtrates. It thus demonstrates that no REE were retained onto the membranes or on the walls of the tubes used.

Sorption behavior was quantified by using the apparent partition coefficient (i.e., Kd), defined as the ratio between REE concentration associated with the biochar and REE concentration in the aqueous solution when the system is at equilibrium, following the equation below:

(1) Kd = (μg REE sorbed/g biochar)/(μg/mL REE in solution)

Rare earth element concentrations in the filtered samples were determined by Inductively Coupled Plasma Mass Spectrometry (ICP-MS; Thermo Fisher Scientific XSERIES2). Quantification was carried out by external calibration (REE multi elemental standard solution from Accu Trace Reference, USA) and using indium (2.5 μg/L) as an internal standard. The instrumental accuracy was assessed by analyzing the SPS-SW2 certified reference material for measurement of elements in surface water (SpectraPur standards, Oslo, Norway). The analyses of real samples were carried out provided that the bias of measured concentration was <5% compared to the certified values.

### 2.3 Modeling

Humic ion-binding Model VI (Tipping, 1998) was introduced into PHREEQC version 3.1.5 (Parkhurst and Appelo, 2013) for the whole REEs group (Marsac et al., 2011). This coupling allows the REE–HA binding description to be improved (Marsac et al., 2011). Indeed, HAs are described as 80 different sites that are expected to participate in the cation-HA binding. To simplify the REE speciation on the HA surface, these 80 sites can be gathered in several groups based on two main characteristics: the chemical nature of the ligands (i.e. carboxylic groups: CG, phenolic groups: PG and carboxy–phenolic groups: CPG) and their denticity (i.e. mono-, bi- and tridentate sites) (Marsac et al., 2011; Tipping, 1998). Modeling parameters are given in Table 1.

**Table 1:** Model VI parameters for humic acid (Tipping, 1998).

Parameter	Description	Values
n_A_	Amount of type A sites (mol g^−1^)	3.3 10^−3^
n_B_	Amount of type B sites (mol g^−1^)	0.5 × n_A_
pK_A_	Intrinsic proton dissociation constant for type A sites	4.1
pK_B_	Intrinsic proton dissociation constant for type B sites	8.8
ΔpK_A_	Distribution terms that modifies pK_A_	2.1
ΔpK_B_	Distribution terms that modifies pK_B_	3.6
log K_MA_	Intrinsic equilibrium constant for metal binding at type A sites	Variable (Marsac et al., 2010)
log K_MB_	Intrinsic equilibrium constant for metal binding at type B sites	3.39 log K_MA_^−^1.15
ΔLK_1_	Distribution term that modifies log K_MA_	2.8 (REE)
ΔLK_2_	Distribution term that modifies the strengths of bidentate and tridentate sites	0.55 log K_NH3_ = 0.29 (REE)
P	Electrostatic parameter	^−^330
K_sel_	Selectivity coefficient for counterion accumulation	1
M	Molecular weight	15000 Da
r	Molecular radius	1.72 nm

## 3 Results and discussion

### 3.1 pH drives the REE sorption on biochar

**Fig. 1:**
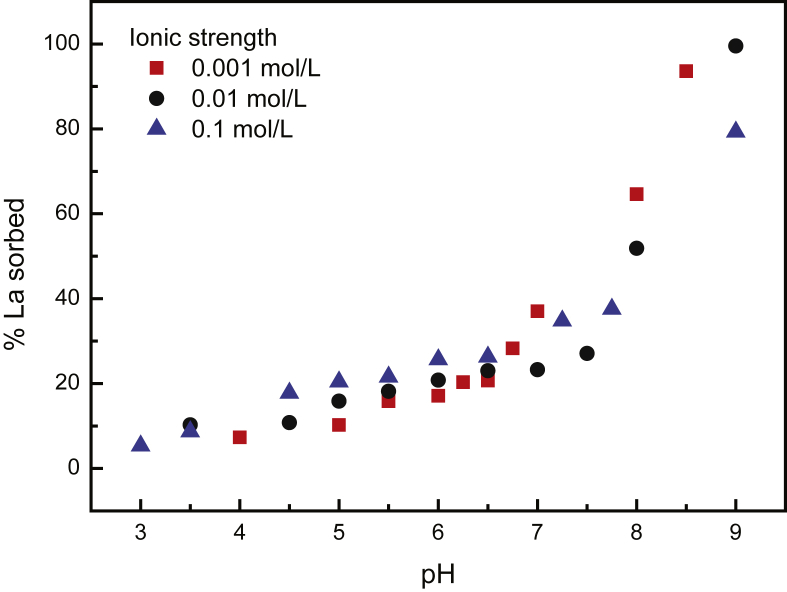
Proportion of La sorbed to biochar as a function of pH for various ionic strength conditions.

The effect of pH on REE sorption was investigated in an initial pH range between 3 and 9. Results are shown for the first REE (i.e., La) in Fig. 1. pH appears to be the parameter which most strongly modified surface reactivity of biochars (Lee et al., 2010). The proportion of REEs removed from solution increases with increasing pH. The removal capacity is minimal at acidic pH (i.e. below pH 5) and increases along with the increase of pH. Irrespective of the ionic strength, Fig. 1 showed a regular increase of La concentration sorbed to the biochar surface with pH. This general feature is well expressed for all REEs and follows general trend of metal sorbed to biochars (Chen et al., 2011; Liu and Zhang, 2009). The REE sorption increase with increasing pH likely results from deprotonation of the biochar surface functional group (e.g., carboxylic) (Harvey et al., 2011; Lu et al., 2012) making them available for REE sorption.

**Fig. 2:**
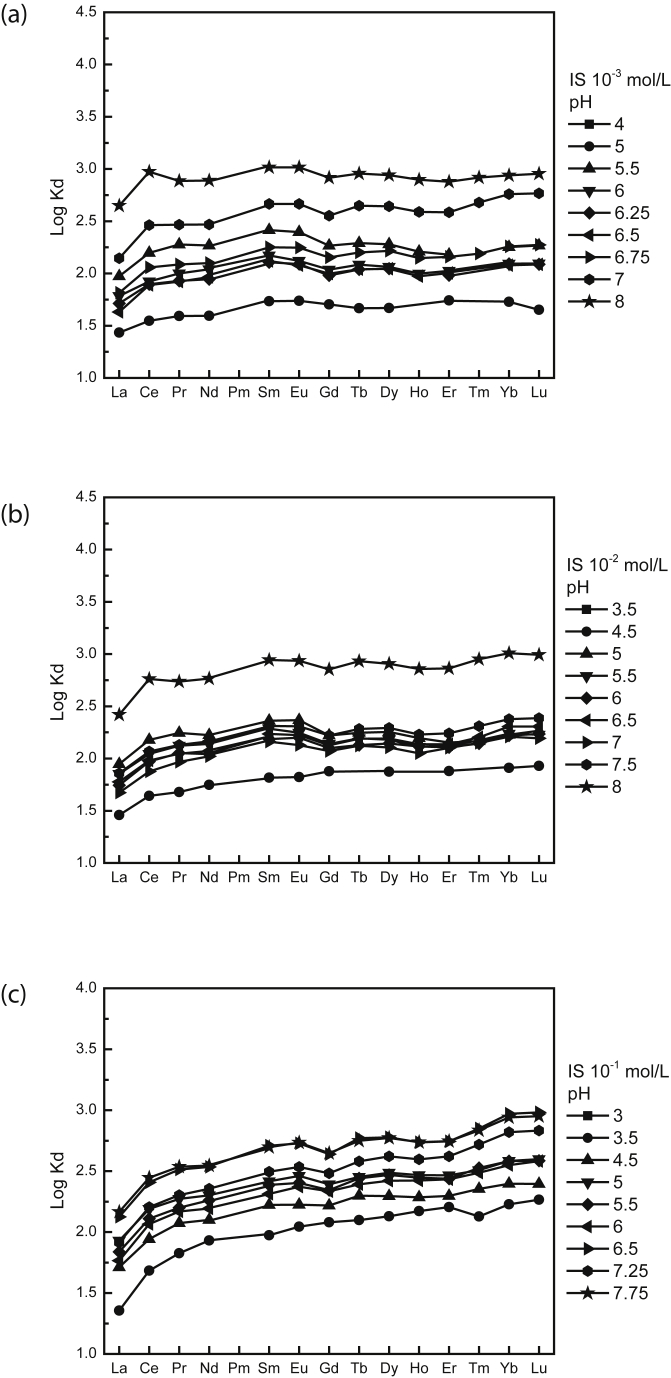
REE patterns for log Kd (REE sorbed onto biochar) for increasing pH values as a function of ionic strength (a) 10^−3^ mol/L, (b) 10^−2^ mol/L and (c) 10^−1^ mol/L.

Moreover, as displayed on Fig. 2, log Kd values increase with pH and log Kd REE patterns highlighted a HREE enrichment (i.e., La/Sm < 1 and Gd/Yb < 1), especially for acidic pH and higher ionic strength. These patterns are in good agreement with the few existing datasets quantifying the binding of REEs with humic substances (Marsac et al., 2010). In particular, the HREE enrichment displayed by REE-biochar complexation pattern determined in this study compares well with results from REE sorption on carboxylic sites hosted by humic acid (HA), fulvic acid (FA) and acetic acid (e.g., Davranche et al., 2015).

### 3.2 Ionic strength effect on REE fractionation

**Fig. 3:**
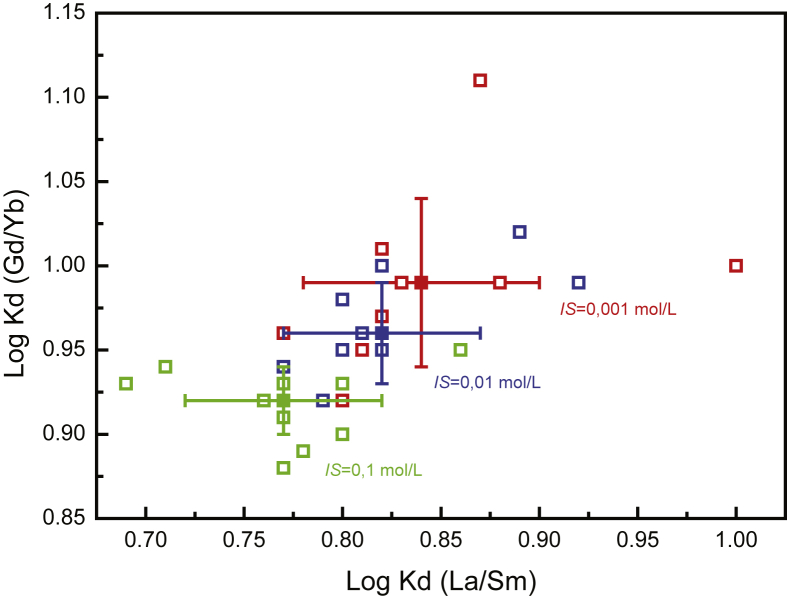
Log Kd (Gd/Yb) versus Log Kd (La/Sm) highlighting change among REE fractionation as a function of ionic strength.

Ionic strength also appeared to have a general effect on the sorption of REEs onto biochar. Log Kd increases with decreasing ionic strength (Fig. 2). However, if we look in details, some exception arises. Indeed, experiments performed at ionic strength of 10^−3^ mol/L and pH 4 and 5 show smaller Kd than that performed at pH 4.5 and ionic strength of 10^−2^ mol/L. In addition, experiment performed at pH 9 and ionic strength of 10^−1^ mol/L shows larger Kd than those performed under ionic strength of 10^−2^ mol/L. Moreover, fractionation between LREEs and HREEs increases with increasing ionic strength (Fig. 3). As shown in Fig. 3, log Kd (Gd/Yb) and log Kd (La/Sm) values vary by ca. 0.15 and 0.2 log units, reflecting fractionation of the REE patterns.

**Fig. 4:**
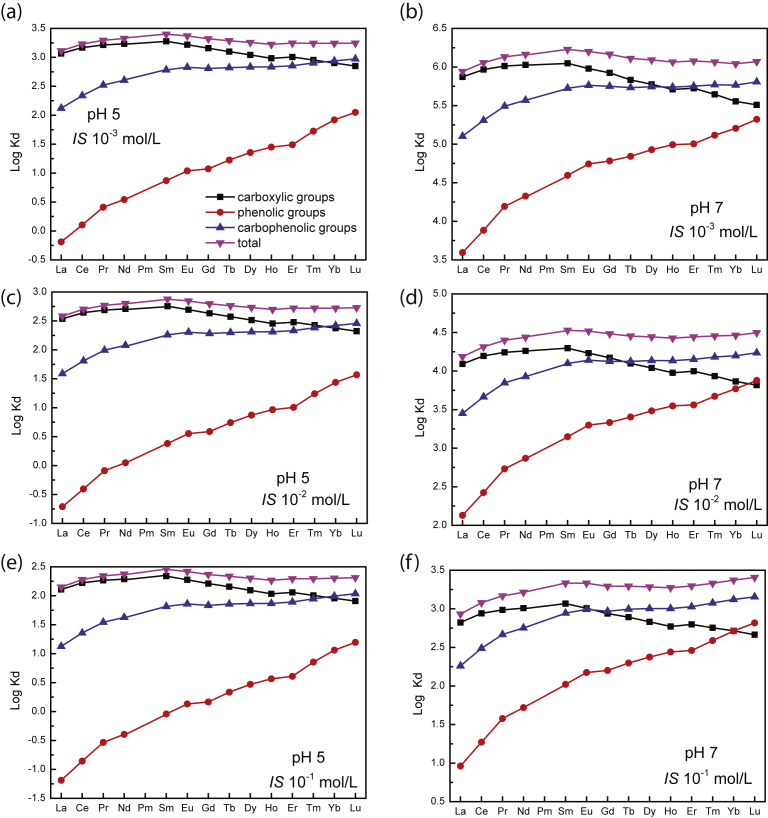
REE patterns of modelled log Kd (REE-humic acid) at (a) pH 5 and *IS* 10^−3^ mol/L and (b) pH pH 7 and *IS* 10^−3^ mol/L, (c) pH 5 and *IS* 10^−2^ mol/L, (d) pH 7 and *IS* 10^−2^ mol/L, (e) pH 5 and *IS* 10^−1^ mol/L and (f) pH 7 and *IS* 10^−1^ mol/L, illustrating evolution of REE fractionation. Modelling was performed using PHREEQC-Model VI considering experimental condition and HA acting as biochar.

Previous studies on organic matter have shown that HREE enrichment patterns is a general feature of simple organic compounds possessing carboxylic functional groups (Marsac et al., 2010). Such carboxylic functional groups can be regarded as weak sites. They displayed log Kd (Gd/Yb) < 1 and log Kd (La/Sm) < 1. A similar comparative approach may provide information about the nature of strong sites that occur at high ionic strength; high affinity sites are generally thought to comprise phenolic, hydroxylic and/or multidentate carboxylic functions (Uchimiya, 2014) displaying log Kd (Gd/Yb) < 1 and log Kd (La/Sm) < 1, illustrating a lanthanide contraction effect. Thus, at low ionic strength (i.e., 10^−3^ mol/L; Figs. 2a and 3), as log Kd (Gd/Yb) is closed to 1, REEs are interpreted to be mainly sorbed by weak carboxylic groups. At high ionic strength (i.e., 10^−1^ mol/L; Figs. 2c and 3), as log Kd (Gd/Yb) decreased below 1, they are interpreted to be mainly bound to strong multidentate carboxylic and phenolic ones (Fig. 4). Moreover, ionic strength dependence can be used to differentiate inner-sphere (ionic strength independent) and outer-sphere (ionic strength dependent) sorption mechanisms (Uchimiya, 2014). An inner sphere complex is a stable molecular entity resulting from the ionic or covalent bonding of a metal ion with a surface functional group. In the outer-sphere mechanism at least one water molecule remains between the surface functional group and sorbed ion (Stumm and Morgan, 1996). Our results reveal thus that the strength of REE bonding with biochar increases with increasing ionic strength. These findings have implications for the use of biochar as a REE immobilizing agent as they indicate that REE immobilization by biochar is expected to be more efficient at high than at low ionic strength at acidic pH whereas at more alkaline pH opposite behavior happened as already observed for other metals (Hadjittofi et al., 2014; Uchimiya, 2014; Vithanage et al., 2015). For example, for La at pH 8, ionic strength increase from 0.001 mol/L to 0.1 mol/L results in removal decrease from 60% to 30% whereas at pH 5, the same ionic strength increase results in removal increase from 10% to 20%. It can be thus inferred that REE binding to biochar was not weak and potentially reversible as regards of pH.

### 3.3 Rare earth elements as a fingerprint

As previously discussed, REE patterns highlight a shift from LREEs to HREEs with increasing ionic strength and pH (Figs. 2 and 3). This suggests that (i) at low ionic strength and acidic pH, REEs are mainly sorbed by carboxylic groups, (ii) at high ionic strength and alkaline pH, REEs are mainly sorbed by carboxy–phenolic groups. Moreover, when cations are bound to biochars, changes may occur in the surface reactivity of the biochars themselves such as (i) the formation of multidentate complexes and (ii) the decrease of the net surface charge (Uchimiya, 2014).

**Fig. 5:**
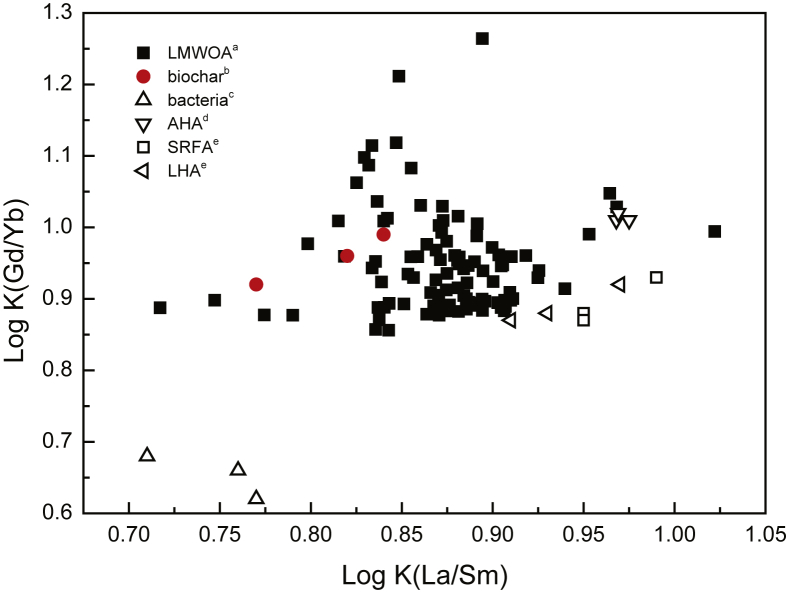
Literature compilation of REE constants with (a) low molecular weight organic acids (Byrne and Li, 1995), (b) biochars from this study (c) *Bacillus subtilis* bacteria (Martinez et al., 2014), (d) Aldrich humic acid (AHA), (e) Suwannee River fulvic acid (SRFA) and Leonardite humic acid (LHA) (Sonke and Salters, 2006).

Previous studies investigating REE binding to humic acids (i.e. considered in this study as analogous material to biochar) reported two kinds of REE–HA patterns depending on the occurring metal loading. (i) At high metal loading, MREE downward concavity patterns are observed (Pourret et al., 2007; Yamamoto et al., 2005). (ii) At much lower metal loading, patterns displaying a lanthanide contraction effect are highlighted (Sonke and Salters, 2006; Stern et al., 2007). The combined effect of metal loading and HA surface heterogeneity (Davranche et al., 2015) is expressed by stronger but less abundant surface sites (i.e., carboxy-phenolic groups) at lower metal loading, and weaker but more abundant surface sites (i.e., carboxylic groups) at higher metal loading. Comparison of experimental log Kd REE-biochar patterns with the pattern for the stability constants (log K) of 101 REE organic ligands (Byrne and Li, 1995) highlight that biochar surface is in the same range as these 101 organic ligands and can thus be considered as a group of discrete sites (Fig. 5). The major sites identified on the biochar surface are the carboxylic and carboxy–phenolic groups (as also highlighted by FTIR), which can form monodentate or multidentate complexes (Uchimiya, 2014). Using this simplified representation, biochar binding properties can be compared with the binding properties of model organic ligands as already shown in literature (Davranche et al., 2015; Marsac et al., 2010, 2011). Indeed, The REE patterns corresponding to the binding of REE with acetate (carboxylic group model ligand) exhibit a MREE downward concavity, whereas NTA (chelate group model ligand) exhibit a lanthanide contraction effect corresponding to both REE-HA patterns obtained at high and low metal loading, respectively. Moreover, as illustrated by the compilation of organic ligands (Byrne and Li, 1995) and infrared spectroscopy results (Gangloff et al., 2014), when the ligand is stronger (i.e., strong aromatic functional groups such as carboxy-phenolic and phenolic groups), HREEs are more strongly bound to the ligand compared to LREEs, as observed on the REE-biochar binding experiments presented in the present study. Moreover, as already proposed by Pourret and Martinez (2009), literature compilation of Log K(La/Sm) organic ligand constants (Byrne and Li, 1995; Martinez et al., 2014; Pourret et al., 2007; Sonke and Salters, 2006) as plotted against Log K(Gd/Yb) (Fig. 5) evidenced that calculated log Kd values in this study are in the range of natural carboxylic acids, further illustrating the major role of carboxylic groups.

To confirm this hypothesis and to gain access to the REE speciation on each biochar site types, the same modeling approach as already performed for humic substances was used (Marsac et al., 2011). Modeling can account for changes in pH and ionic strength as already proposed by Alam et al. (2017) for cadmium and selenium. Considering biochar as analogous to humic substances, further allows to dissociate binding parameter of carboxylic and carboxy–phenolic sites. Such modeling also allows accessing the REE distribution onto each binding group (Fig. 4; Table 2). At low ionic strength (i.e. 10^−3^ mol/L) and pH 3, REEs are mostly bound to carboxylic groups (33% for La) whereas at higher ionic strength and pH 3, LREEs are bound to carboxylic groups (21%) and HREEs are bound to carboxylic (13% for Lu) and carboxy-phenolic (10 % for Lu) groups. At pH of 5 and irrespective of the ionic strength, LREEs are mostly bound to carboxylic (from 83% at 10^−3^ mol/L to 57% at 10^−1^ mol/L for La) whereas HREEs are bound to carboxylic (from 38% at 10^−3^ mol/L to 28% at 10^−1^ mol/L for Lu) and carboxy-phenolic (from 50% at 10^−3^ mol/L to 38% at 10^−1^ mol/L for Lu) groups. At a near neutral pH, LREEs are bound to carboxylic groups (from 85% at 10^−3^ mol/L to 71% at 10^−1^ mol/L for La) and carboxy-phenolic groups (from 14% at 10^−3^ mol/L to 19% at 10^−1^ mol/L for La) and HREEs via carboxy–phenolic (from 55% at 10^−3^ mol/L to 54% at 10^−1^ mol/L for Lu), carboxylic (from 28% at 10^−3^ mol/L to 18% at 10^−1^ mol/L for Lu) and phenolic (from 18% at 10^−3^ mol/L to 25% at 10^−1^ mol/L for Lu) groups (Fig. 4; Table 2). As already discussed, modeling further indicates that REE immobilization by biochar is expected to be more efficient at high than at low ionic strength at acidic pH whereas at more alkaline pH opposite behavior happened as already observed for other metals (Hadjittofi et al., 2014; Uchimiya, 2014; Vithanage et al., 2015). It must be noted that modeling considering HA overestimates sorption of REE at pH 7 but overall is a good representation of experimental results. Eventually, the denticity of the complex is dependent on the metal loading. The number of groups coordinated to REEs increased with decreasing loading (Marsac et al., 2011; 2015). The binding of REEs to blocked phenolic and carboxylic sites of HA suggests that REE binding to stronger sites can be assumed to be the chelator (Kautenburger et al., 2014). They consist in a combination of phenolic and carboxylic groups (Kautenburger et al., 2014). EXAFS results considering ytterbium complexation with HA synthesized at low and high metal loadings, show that at low metal loading, REEs are bound to HA through bi-ligand complexes without any chelation effect. In these conditions, REE act as a cation bridge between two organic molecules. At the opposite, at high metal loading, REEs are bound to HA via multi-carboxylic ligands (Marsac et al., 2015). This modelling approach is a considerable advance in the sense that such modeling considering HA surfaces as analogous of biochar surfaces account for changes in pH, ionic strength, and metal-to-sorbent ratio.

**Table 2:** Proportions of REE sorbed by carboxylic groups (CG), phenolic groups (PG), carboxy-phenolic groups (CPG) and total (TOT) for various experimental conditions obtained by modeling using PHREEQC-Model VI.

IS	%CG	%PG	%CPG	%TOT	%CG	%PG	%CPG	%TOT	%CG	%PG	%CPG	%TOT
	0.001	0.001	0.001	0.001	0.01	0.01	0.01	0.01	0.1	0.1	0.1	0.1
pH	3	3	3	3	3	3	3	3	3	3	3	3
La	33	0	1	34	30	0	1	31	21	0	1	22
Ce	39	0	2	40	35	0	1	37	26	0	1	27
Pr	41	0	3	43	37	0	2	40	28	0	2	30
Nd	42	0	3	45	38	0	3	41	29	0	2	31
Sm	44	0	4	48	40	0	4	44	31	0	4	35
Eu	40	0	5	45	37	0	5	42	28	0	4	32
Gd	37	0	5	42	33	0	5	38	25	0	4	29
Tb	33	0	6	39	30	0	5	36	23	0	5	27
Dy	30	0	7	37	27	0	6	34	20	0	5	25
Ho	27	0	7	35	25	0	6	31	18	0	5	23
Er	28	1	7	36	26	0	7	33	19	0	6	25
Tm	25	1	10	36	23	1	9	32	17	1	7	25
Yb	22	2	11	36	20	2	11	32	15	1	9	25
Lu	20	2	13	36	18	2	12	32	13	1	10	25

IS	%CG	%PG	%CPG	%TOT	%CG	%PG	%CPG	%TOT	%CG	%PG	%CPG	%TOT
	0.001	0.001	0.001	0.001	0.01	0.01	0.01	0.01	0.1	0.1	0.1	0.1
pH	5	5	5	5	5	5	5	5	5	5	5	5
La	83	0	9	92	74	0	8	82	57	0	6	63
Ce	82	0	12	94	75	0	11	86	61	0	8	70
Pr	79	0	16	95	73	0	15	88	61	0	11	73
Nd	77	0	18	95	71	0	17	88	61	0	13	74
Sm	72	0	23	96	68	0	22	90	59	0	18	77
Eu	68	0	28	96	63	0	26	89	55	0	21	76
Gd	65	1	29	95	61	1	27	88	52	0	22	74
Tb	61	1	33	95	56	1	30	87	48	1	24	72
Dy	58	1	36	94	53	1	33	87	44	1	26	71
Ho	54	2	38	94	49	2	35	86	40	1	27	69
Er	54	2	38	94	49	2	35	86	41	1	28	70
Tm	48	3	43	94	44	3	40	86	36	3	32	70
Yb	43	4	47	94	39	4	43	86	32	4	35	71
Lu	38	6	50	94	34	6	46	86	28	5	38	71

IS	%CG	%PG	%CPG	%TOT	%CG	%PG	%CPG	%TOT	%CG	%PG	%CPG	%TOT
	0.001	0.001	0.001	0.001	0.01	0.01	0.01	0.01	0.1	0.1	0.1	0.1
pH	7	7	7	7	7	7	7	7	7	7	7	7
La	85	0	14	100	80	1	18	99	71	1	19	91
Ce	81	1	18	100	76	1	22	100	68	1	24	93
Pr	76	1	23	100	70	2	28	100	62	2	30	95
Nd	73	1	25	100	66	3	31	100	59	3	33	95
Sm	66	2	31	100	59	4	37	100	52	5	39	96
Eu	60	3	36	100	52	6	42	100	46	7	44	96
Gd	57	4	38	100	49	7	44	100	43	8	46	96
Tb	53	5	42	100	44	9	47	100	38	10	48	96
Dy	48	7	45	100	39	11	49	100	34	12	50	96
Ho	44	8	47	100	36	13	51	100	30	14	52	96
Er	44	8	47	100	36	13	51	100	30	14	52	96
Tm	38	11	51	100	30	16	53	100	25	17	53	96
Yb	33	15	53	100	25	20	54	100	21	21	54	97
Lu	28	18	55	100	21	24	55	100	18	25	54	97

### 3.4 Implications for the use of biochar as a sorbent for metals

An increasing number of studies consider biochar as a potential sorbent to remove and immobilize metals in water and soil, respectively (Ahmad et al., 2014). However, little is known concerning the stability, and therefore the longevity, of the immobilizing processes when environmental parameters change (Houben et al., 2013b). Like any other organic matter, biochar can possess functional groups, namely carboxyl and phenolic groups (Uchimiya et al., 2011), which host low and high metal affinity sites, respectively. Here, our results showed that, despite a decrease in the amount of metal sorbed onto biochar, increasing ionic strength favors metal sorption on high affinity sites, as indicated by the higher relative contribution of carboxy-phenolic and phenolic groups. Moreover, our data demonstrated that rising pH from acidic to near neutral values increases both the amount of sorbed metal and the relative contribution of carboxy-phenolic and phenolic groups to metal sorption. These findings have great implications for the use of biochar as a sorbent or an immobilizing agent for metals in water and soil because they reveal that environmental conditions affect not only the amount of sorbed metal onto biochar but also the metal-biochar binding strengths and stabilities. Overall, the strength of the binding of metals with biochar increases when pH and ionic strength increase, indicating that biochar is more efficient for long-term metal immobilization at near neutral pH and high ionic strength. Eventually, considering surface complexation modeling of biochar comparable to humic substances is a promising direction even if some other alternative may be considered (Alam et al., 2017).

## 4 Conclusions

The efficiency of biochar to sorb metals is critically dependent on the type of metal-binding sites at the surface of biochar particles. Although techniques such as FTIR provide a preliminary biochar characterization by quantifying these sites, they give no information about the change in surface reactivity of biochar once added to water or soil. Using a new approach based on previous works on REE interactions with humic substances, this study is, to our knowledge, the first attempting to elucidate the relative contribution of these binding sites to metal sorption onto biochar under various conditions (i.e. various pH and ionic strengths). Our findings indicate that environmental conditions affect not only the amount of sorbed metal onto biochar but also the metal-biochar binding strengths and stabilities. In particular, the strength of the metal binding with biochar increases when pH and ionic strength increase, which has a great implication for the use of biochar as a metal sorbent in the long run. More generally, our study highlights the great potential of REEs as a fingerprint for the characterization of metal binding sites onto sorbing matrixes, including biochar.

## Declarations

### Author contribution statement

Olivier Pourret, David Houben: Conceived and designed the experiments; Performed the experiments; Analyzed and interpreted the data; Contributed reagents, materials, analysis tools or data; Wrote the paper.

### Funding statement

This research did not receive any specific grant from funding agencies in the public, commercial, or not-for-profit sectors.

### Competing interest statement

The authors declare no conflict of interest.

### Additional information

No additional information is available for this paper.
